# Prioritization of Osteoporosis‐Associated Genome‐wide Association Study (GWAS) Single‐Nucleotide Polymorphisms (SNPs) Using Epigenomics and Transcriptomics

**DOI:** 10.1002/jbm4.10481

**Published:** 2021-03-19

**Authors:** Xiao Zhang, Hong‐Wen Deng, Hui Shen, Melanie Ehrlich

**Affiliations:** ^1^ Tulane Center for Biomedical Informatics and Genomics, Deming Department of Medicine, School of Medicine Tulane University New Orleans LA USA; ^2^ Tulane Cancer Center and Hayward Genetics Center Tulane University New Orleans LA USA

**Keywords:** EPIGENETICS, MOLECULAR PATHWAYS – REMODELING, OSTEOBLASTS, OSTEOPOROSIS, Wnt/β‐CATENIN

## Abstract

Genetic risk factors for osteoporosis, a prevalent disease associated with aging, have been examined in many genome‐wide association studies (GWASs). A major challenge is to prioritize transcription‐regulatory GWAS‐derived variants that are likely to be functional. Given the critical role of epigenetics in gene regulation, we have used an unusual epigenetics‐based and transcription‐based approach to identify some of the credible regulatory single‐nucleotide polymorphisms (SNPs) relevant to osteoporosis from 38 reported bone mineral density (BMD) GWASs. Using Roadmap databases, we prioritized SNPs based upon their overlap with strong enhancer or promoter chromatin preferentially in osteoblasts relative to 12 heterologous cell culture types. We also required that these SNPs overlap open chromatin (Deoxyribonuclease I [DNaseI]‐hypersensitive sites) and DNA sequences predicted to bind to osteoblast‐relevant transcription factors in an allele‐specific manner. From >50,000 GWAS‐derived SNPs, we identified 14 novel and credible regulatory SNPs (Tier‐1 SNPs) for osteoporosis risk. Their associated genes, *BICC1*, *LGR4*, *DAAM2*, *NPR3*, or *HMGA2*, are involved in osteoblastogenesis or bone homeostasis and regulate cell signaling or enhancer function. Four of these genes are preferentially expressed in osteoblasts. *BICC1*, *LGR4*, and *DAAM2* play important roles in canonical Wnt signaling, a pathway critical for bone formation and repair. The transcription factors predicted to bind to the Tier‐1 SNP‐containing DNA sequences also have bone‐related functions. We present evidence that some of the Tier‐1 SNPs exert their effects on BMD risk indirectly through little‐studied long noncoding RNA (lncRNA) genes, which may, in turn, control the nearby bone‐related protein‐encoding gene. Our study illustrates a method to identify novel BMD‐related causal regulatory SNPs for future study and to prioritize candidate regulatory GWAS‐derived SNPs, in general. © 2021 The Authors. *JBMR Plus* published by Wiley Periodicals LLC on behalf of American Society for Bone and Mineral Research.

## 1 Introduction

Osteoporosis, which affects about 200 million people worldwide, involves a loss of bone mass, strength, and normal microarchitecture.^(^ ^1^ ^)^ Both osteoporosis and bone fracture increase greatly with age.^(^ ^2^ ^)^ Bone mineral density (BMD) of the hip, spine, limb, or total body is the quantitative trait used most frequently to detect or predict osteoporosis.^(^ ^1^, ^3^ ^)^ In the clinic, BMD is usually determined by dual‐energy x‐ray absorptiometry (DXA). In genome‐wide association studies (GWASs), the simpler‐to‐perform ultrasound‐measured heel bone density (estimated BMD [eBMD]) is often used. Like DXA‐determined BMD, eBMD has a high heritability (50–80%) and can predict bone fracture, after correcting for the effects of height, weight, age, and sex.^(^ ^4^ ^)^ Even low‐frequency variants (minor allele frequency of 1–5%) or rare variants may contribute to decreased BMD and increased bone fracture.^(^ ^5^ ^)^

From DXA or eBMD GWAS (collectively referred to here as BMD GWAS), hundreds of genes have been identified as having nearby or overlapping genetic variants significantly associated with osteoporosis risk.^(^ ^1^, ^4^, ^6^ ^)^ Most BMD or other trait‐associated variants are located in noncoding regions of the genome.^(^ ^1^, ^7^ ^)^ Identifying causal osteoporosis‐risk genes and their associated variants, usually single‐nucleotide polymorphisms (SNPs), is challenging for variants that are not missense coding variants. The GWAS‐derived SNP exhibiting the smallest *p* value for association with the studied trait at a given locus (index SNP) is often linked to very many proxy SNPs solely because of linkage disequilibrium (LD) rather than because of biological relevance. Recently, whole‐genome profiles of epigenetic features, such as overlap with open chromatin (e.g., deoxyribonuclease I [DNaseI] hypersensitive sites [DHSs]), have been used to help discriminate likely causal BMD regulatory variants from bystander variants in high LD with them.^(^ ^5^, ^6^, ^8^, ^9^, ^10^ ^)^ Moreover, GWAS SNPs are generally also enriched in enhancer or promoter chromatin.^(^ ^7^, ^10^ ^)^ The importance of considering such epigenetic features at BMD GWAS‐derived SNPs is also evidenced by the finding that specific epigenetic changes play a key role in bone formation and remodeling.^(^ ^11^, ^12^ ^)^

Using epigenetics to help prioritize BMD GWAS variants requires choosing the cell types for study. Bone has one of the most complex developmental and repair pathways due in part to its rigidity, strength, highly dynamic nature, and multiple structural and physiological roles.^(^ ^13^ ^)^ Osteoblasts (ostb) play a central role in both intramembranous ossification and in the more widespread and complicated endochondral ossification pathway for bone formation based upon chondrocytes (chond).^(^ ^11^ ^)^ Surprisingly, there is evidence for hypertrophic chond transdifferentiating to ostb, which is consistent with the considerable overlap of transcription profiles of these two morphologically dissimilar bone cell types.^(^ ^14^, ^15^ ^)^ Ostb and chond are derived from a heterogeneous mixture of bone‐derived stem or stem‐like cells commonly referred to as mesenchymal stem cells (MSCs).^(^ ^13^ ^)^ Bone‐destroying osteoclasts, which arise from the monocyte blood lineage, contribute to osteoporosis when bone resorption is excessive. Remodeling of cortical bone occurs through complex multicellular units containing ostb and osteoclasts, which modulate each other's function and are often responsive to some of the same growth factors and cytokines, although sometimes with different responses.^(^ ^2^, ^16^ ^)^ Osteocytes (terminally differentiated ostb), which constitute most of the cells in cortical bone, coordinate the differentiation and activity of ostb and osteoclasts in response to mechanical load or injury.^(^ ^17^ ^)^

Because of the centrality of ostb to normal bone homeostasis and the availability of epigenomic and transcriptomic profiles for comparing human cell cultures of ostb, chond, MSC, and heterologous primary cell cultures, we focused on examining the epigenetics of BMD GWAS‐derived SNPs in ostb and, secondarily, in chond and MSCs. In a recent study, we used epigenetics to prioritize best‐candidate causal regulatory variants for BMD GWAS SNPs and obesity GWAS SNPs at a single gene, *TBX15*, which encodes a limb development‐associated transcription factor (TF).^(^ ^18^ ^)^ In the present study, we take the unusual approach of looking genome‐wide not only for SNPs overlaying enhancer or promoter chromatin in ostb but also for such SNPs that displayed these types of regulatory chromatin preferentially in ostb relative to many types of cell cultures not directly related to bone. Using epigenomics, transcriptomics, gene ontology (GO) analysis, and TF binding site (TFBS) prediction in an analysis of whole‐genome data from 38 BMD GWAS, we identified 14 high‐priority candidates for causal regulatory SNPs associated with five bone‐relevant genes (*BICC1*, *NPR3*, *LGR4*, *HMGA2*, and *DAAM2*). None of these SNPs was previously described as associated with osteoporosis risk.

## 2 Materials and Methods

### 2.1 BMD GWAS‐derived SNPs

Index SNPs associated with BMD (*p* < 5 × 10^−8^) were retrieved from 38 studies in the National Human Genome Research Institute–European Bioinformatics Institute (NHGRI‐EBI) GWAS Catalog^(^ ^19^ ^)^ (downloaded October 2019; Supplementary Table S1). We expanded the index SNPs to a set of proxy SNPs (*r* ^2^ ≥ 0.8, EUR from the 1000 Genome Project Phase 3^(^ ^20^ ^)^) by using PLINK v1.9^(^ ^21^ ^)^ (https://www.cog-genomics.org/plink/1.9/) and/or LDlink v3.9^(^ ^22^ ^)^ (https://ldlink.nci.nih.gov/). For the five best candidate genes, we also extracted imputed SNPs (*p* < 5 × 10^−8^) for total‐body DXA BMD^(^ ^3^ ^)^ and (*p* < 6.6 × 10^−9^) for eBMD^(^ ^6^ ^)^ to look for additional Tier‐1 SNPs.

### 2.2 Transcriptomics

For determining preferential expression of genes in ostb we obtained the reads per kilobase million (RPKM) for ostb and 11 heterologous cell cultures, not including MSC or chond, from the ENCODE RNA‐seq database (Supplementary Methods; https://genome.ucsc.edu/cgi-bin/hgFileUi?db=hg19&g=wgEncodeCshlLongRnaSeq). Preferential expression was defined as the ratio of ostb RPKM/median non‐ostb RPKM >5 and with a minimum of RPKM >1 for ostb. The differentiation status of the commercially obtained ostb that had been used to generate the ENCODE RNA‐seq data as well as for the epigenomic data (see below) is unclear. However, expression and chromatin segmentation profiles for *SP7*, *RUNX2*, *SPP1*, *IBSP*, *TNFRSF11B*, *BGLAP*, and *ALPL* indicate selective transcription of middle and late ostb differentiation marker genes by ostb samples and chond‐specific expression of *SOX5* in chond samples (Supplementary Figs. S1 and S2). For some genes, we also examined tissue expression profiles (median transcripts per million [TPM], and tissue expression QTL [eQTL]) from the GTEx database^(^ ^23^ ^)^ (https://www.gtexportal.org/) and mouse expression microarray profiles from BioGPS^(^ ^24^ ^)^ (http://biogps.org/).

### 2.3 Epigenomics

Chromatin state segmentation data for the 15 examined cell culture types (Supplementary Methods and Table S3), including MSC and chond, were obtained from the 18‐state Roadmap model with strong promoter chromatin as state 1 and strong enhancer chromatin as states 3, 8, or 9 (Roadmap^(^ ^7^ ^)^; https://egg2.wustl.edu/roadmap/data/byFileType/chromhmmSegmentations/ChmmModels/core_K27ac/jointModel/final/). In figures, for clarity in depicting these data, we slightly modified the color‐coding of the chromatin state segmentation as indicated. DHS and peaks of histone H3 lysine‐27 acetylation (H3K27ac) were defined as narrowPeaks as determined by Roadmap^(^ ^7^ ^)^ (https://egg2.wustl.edu/roadmap/data/byFileType/peaks/consolidatedImputed/narrowPeak).

### 2.4 Predictions of allele‐specific transcription factor binding sites and analyses of gene functional terms

Predictions of allele‐specific TFBS were made using a 21‐base sequence centered around the SNP of interest for comparison to the TRANSFAC v2019.3 database (see Supplemental Methods for more details). We checked that each TF for a matching TFBS had appreciable expression in ostb (RPKM ≥0.8). For determining that a TFBS overlapping a SNP is likely to be allele‐specific in its TF binding, we required the difference in position probability between the reference (Ref) and alternative (Alt) alleles to be greater than fivefold in the position weight matrix (PWM). In addition, we used manual curation to retain only those TRANSFAC TFBS predictions for which all the conserved positions had exact matches to the SNP‐containing sequence and no more than one base in a partly conserved position had only a partial match (at least 20% as good as the best PWM match). DAVID v6.7^(^ ^25^ ^)^ (https://david.ncifcrf.gov/tools.jsp) was used for functional classification of reference genes and GREAT v4.0.4^(^ ^26^ ^)^ (http://great.stanford.edu/) for prioritization of genes linked to our epigenetically‐selected GWAS SNP subset.

### 2.5 Fine‐mapping analyses

We applied a Bayesian approach, Probabilistic Annotation INtegraTOR (PAINTOR, version 3.1)^(^ ^27^ ^)^ using local significant SNPs (*p* < 6.6 × 10^−9^) obtained from eBMD summary data^(^ ^6^ ^)^ in the 1‐megabase (1‐Mb) region extending from each of the ends of our best candidate regulatory genes. The 1000 Genome Project Phase 3 (European ancestry, EUR)^(^ ^20^ ^)^ served as the reference genotypes. For functional annotations, we integrated overlap of the SNPs with the following attributes: Roadmap‐derived enhancer and promoter chromatin segmentation states (1, 3, 8, and 9), and narrow peaks for DHS and H3K27ac in ostb, and exonic DNA.

## 3 Results

### 3.1 Prioritization of 14 SNPs as candidate causal regulatory SNPs (Tier‐1 SNPs) from 38 BMD GWAS

**Fig 1:**
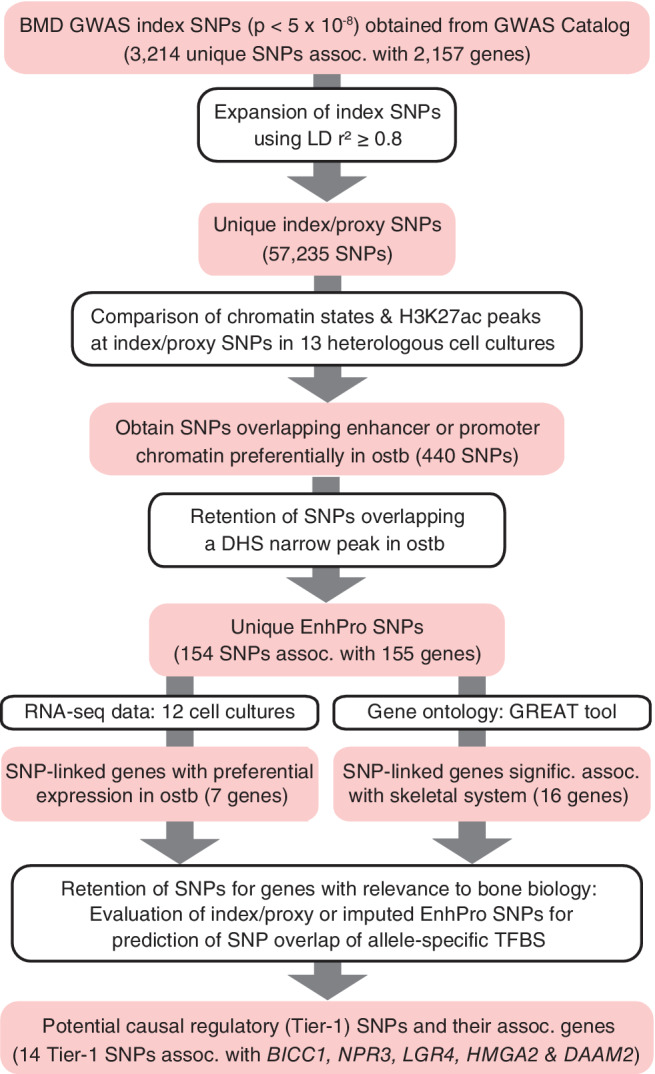
Workflow for prioritizing 14 potential causal regulatory (Tier‐1) SNPs from 38 BMD GWAS. Summary of prioritization of plausible regulatory BMD SNPs. Abbreviations: Assoc, associated; BMD, bone mineral density; DHS, deoxyribonuclease I hypersensitive site; GREAT tool, http://great.stanford.edu/public/html/; GWAS, genome‐wide association study; H3K27ac, histone H3 lysine‐27 acetylation (an epigenetic mark of active enhancers or promoters); ostb, osteoblasts; SNP, single‐nucleotide polymorphism; TFBS, transcription factor binding site.

To identify high‐priority candidates for causative regulatory variants from BMD GWAS, we obtained index SNPs from 38 eBMD or DXA BMD studies (Fig. 1; Supplementary Table S1) and expanded them (LD threshold of *r* ^2^ ≥ 0.8) using the EUR population because it was the predominant one studied. As expected, the GO term with the most enrichment among the 2157 reference (reported/mapped) genes associated with the BMD GWAS index SNPs was skeletal system development (Supplementary Table S2). In accord with other analyses,^(^ ^1^, ^7^ ^)^ only a small percentage (2%, 1139 SNPs) of the 57,235 BMD‐related index/proxy SNPs mapped to coding regions, and they were associated with 29% of the reference genes.

To prioritize BMD GWAS regulatory SNPs, we set as the initial criterion that the SNPs had to overlap bone‐cell specific epigenetics tags to maximize the likelihood that the SNPs specifically affect transcription in the skeletal system (Fig. 1). Our protocol was not meant to be all‐inclusive but rather to identify some highly credible risk alleles. The epigenetics of ostb, but not osteoclasts or osteocytes, was studied because only ostb had available whole‐genome epigenetic profiles. The first epigenetic tag we looked for was strong transcription‐regulatory enhancer or promoter chromatin in ostb irrespective of genomic position. Promoter or enhancer chromatin states had been determined from genome‐wide profiles of histone H3 K4 trimethylation (H3K4me3) and H3K27ac for promoter chromatin and H3K4me1 and H3K27ac for enhancer chromatin.^(^ ^7^ ^)^ We found that only ~0.8% (440) of the 57,235 index/proxy SNPs were preferentially associated with promoter or enhancer chromatin in ostb and, in addition, overlapped a narrow peak of H3K27ac in this cell type (Fig. 1; data not shown). Preferential overlap of positive‐regulatory chromatin in ostb was defined as such overlap in ostb but in no more than three of the following 12 cell cultures from the Roadmap database^(^ ^7^ ^)^: adult skin fibroblasts (fib), adult or fetal lung fib, foreskin or dermal keratinocytes, foreskin melanocytes, astrocytes, umbilical cord endothelial cells, myoblasts (myob), mammary epithelial cells (HMEC), embryonic stem cells, and a lymphoblastoid cell line. Subsequent filtering for overlap of open chromatin (DHS) in ostb gave 154 SNPs, which are referred to as EnhPro SNPs (GWAS‐derived SNPs at Enhancer or Promoter chromatin seen preferentially in ostb and at an ostb DHS; Supplementary Table S3). These 154 EnhPro SNPs were associated with 174 index SNPs and 156 reference genes obtained from the GWAS Catalog.^(^ ^19^ ^)^ EnhPro SNPs also often overlapped strong enhancer or promoter chromatin in bone marrow‐derived MSCs (74%) or in chond (79%), but not in monocytes (16%). We also searched for overlap of GWAS derived SNPs with binding sites for the looping protein CCCTC‐binding factor (CTCF) but found very few sites that bound this protein preferentially in ostb (data not shown), and almost two orders of magnitude more sites that overlapped promoter or enhancer chromatin preferentially in ostb.

To identify EnhPro SNPs most likely to contribute to inherited osteoporosis risk, we used two alternate methods to evaluate their associated genes (Fig. 1). First, we identified seven EnhPro SNP‐associated genes, *RUNX2*, *TBX15*, *ADAM12*, *NPR3*, *BICC1*, *LGR4*, and *SPECC1*, that were preferentially expressed in ostb, which we defined as greater than five times more RPKM in ostb than in the median of 11 heterologous cell types and an RPKM >1 in ostb (Supplementary Table S4). All but *SPECC1* have known bone‐related functions.^(^ ^11^, ^18^, ^28^, ^29^, ^30^, ^31^ ^)^ Because we recently analyzed EnhPro SNPs for *TBX15*,^(^ ^18^ ^)^ we eliminated it as well as *SPECC1* from further consideration. The second method we used to identify EnhPro SNP‐associated genes relevant specifically to bone biology was to examine these genes for bone biology‐related GO terms, literature references for their skeletal association, and appreciable expression in ostb, chond, or MSC. This alternative search gave seven additional EnhPro SNP‐associated genes, *HMGA2*, *BMP5*, *DLX6*, *SIX1*, *SMAD3*, *TRPS1*, and *WNT7B* (Supplementary Table S5). The EnhPro SNPs from these prioritized genes were examined for predicted overlapping allele‐specific TFBS using the TRANSFAC database and stringent hand curation. We found that four of these genes, *BICC1*, *NPR3*, *LGR4*, and *HMGA2*, had strong predictions of allele‐specific TFBS overlapping at least one of their EnhPro SNPs and refer to these SNPs as Tier‐1 SNPs. The fifth and last Tier‐1 SNP‐associated gene, *DAAM2*, was found using a slightly relaxed prioritization procedure for identification of EnhPro SNPs, as described in Section 3.6. In addition to the 11 index/proxy‐derived Tier‐1 SNPs, we found three more Tier‐1 SNPs by examining imputed total body‐BMD^(^ ^3^ ^)^ and eBMD^(^ ^6^ ^)^ SNPs associated with these five prioritized genes (Table 1).

**Table 1:** Fourteen Prioritized Candidates for Transcription‐Regulatory BMD‐Causal Variants (Tier‐1 SNPs) in Five Bone‐Related Genes

Gene	Tier‐1 SNP for BMD association	I/P or Imp SNP	Distance of SNP to gene TSS (kb)	Alleles (Ref/Alt)	BMD‐increasing allele (Freq EUR)^a^	Chromatin state^b^ at the SNP in ostb	Predicted TF binding^c^	
							Ref allele	Alt allele
BICC1	rs112597538	I/P	−0.1	T/C	Unknown (0.50)	Str prom	SREBF1/2	None
	rs1896245	I/P, Imp	3.2	T/G	Alt (0.50)	Str enh/prom	SATB1	None
	rs1896243	I/P, Imp	3.5	C/T	Alt (0.50)	Str enh	None	TCF3
	rs11006188	Imp	60.6	G/C	Ref (0.60)	Str enh	SMAD, GLIS3	NR2F6
	rs1982173	Imp	61.2	G/T	Ref (0.60)	Str enh	RBPJ	None
NPR3	rs1173771	I/P	104.3	A/G	Alt (0.58)	Str enh	SATB1	None
	rs7733331	I/P	118.1	T/C	Alt (0.58)	Str enh	GTF2I	None
LGR4	rs10835153	I/P, Imp	−181	A/T	Ref (0.41)	Str enh	None	BPTF
HMGA2	rs80019710	Imp	−231.1	C/T	Alt (0.05)	Str enh	None	HMGA1
	rs17101510	I/P, Imp	−231	C/T	Alt (0.02)	Str enh	None	YY1
	rs12296417	I/P, Imp	−230.5	T/A	Alt (0.02)	Str enh	None	SATB1, ZBTB20
DAAM2	rs2504105	I/P, Imp	62.4	A/G	Ref (0.40)	Str enh/prom	SMAD	None
	rs2504104	I/P, Imp	62.4	A/G	Ref (0.40)	Str enh/prom	None	RUNX2/1, CBFB
	rs2504103	I/P, Imp	62.5	C/G	Ref (0.40)	Str enh/prom	None	NFIC

*Note*: 14 Tier‐1 SNPs (prioritized candidates for regulatory osteoporosis‐risk SNPs) and their five associated genes determined from 38 BMD GWAS.

Abbreviations: Alt, alternate allele; BMD, bone mineral density; GWAS, genome‐wide association study; I/P, index or proxy SNP; Imp, imputed SNP; ostb, osteoblasts; Ref, reference allele; SNP, single‐nucleotide polymorphism; Str enh, strong enhancer chromatin; Str prom, strong promoter chromatin; TF, transcription factor; TSS, transcription start site.

^a^ The BMD‐increasing alleles and their frequency in the European population.

^b^ From Roadmap 18‐State/Auxiliary Hidden Markov Model, States 1, 3, 8, or 9.

^c^ Predictions of allele‐specific TF binding sites by hand curation of data from the TRANSFAC database and database‐verification of TF expression in ostb.

### 3.2 *BICC1* has five BMD‐risk Tier‐1 SNPs near the 5′ end of the gene

Among the five prioritized genes, BicC family RNA binding protein 1 (*BICC1*) had the most Tier‐1 SNPs (five; Table 1)*. BICC1* encodes a translation‐regulatory protein that is implicated in osteoblastogenesis, plays critical roles in several signal transduction pathways, and is required for development and homeostasis of various organs including bone.^(^ ^28^, ^32^, ^33^ ^)^ It is the only gene in the surrounding 1‐Mb region that is preferentially and appreciably expressed in ostb and is expressed more strongly in skin fib than in a variety of tissues although more weakly in skin fib than in ostb (Supplementary Figs. S3 and S4). Mouse expression microarray profiles indicate that there is much transcription of *Bicc1* in ostb and negligible expression in osteoclasts, as was the case for all the other Tier‐1 SNP‐associated genes (Supplementary Figs. S5*A*,*B* and S6). As positive controls, we checked that osteoclast markers *Ctsk*, and *Tnfrsf11a/Rank* exhibit the expected osteoclast‐associated expression on these microarrays (Supplementary Fig. S5*C*,*D*). We conclude that the Tier‐1 SNPs that we identified are unlikely to modulate transcription in osteoclasts.

**Fig 2:**
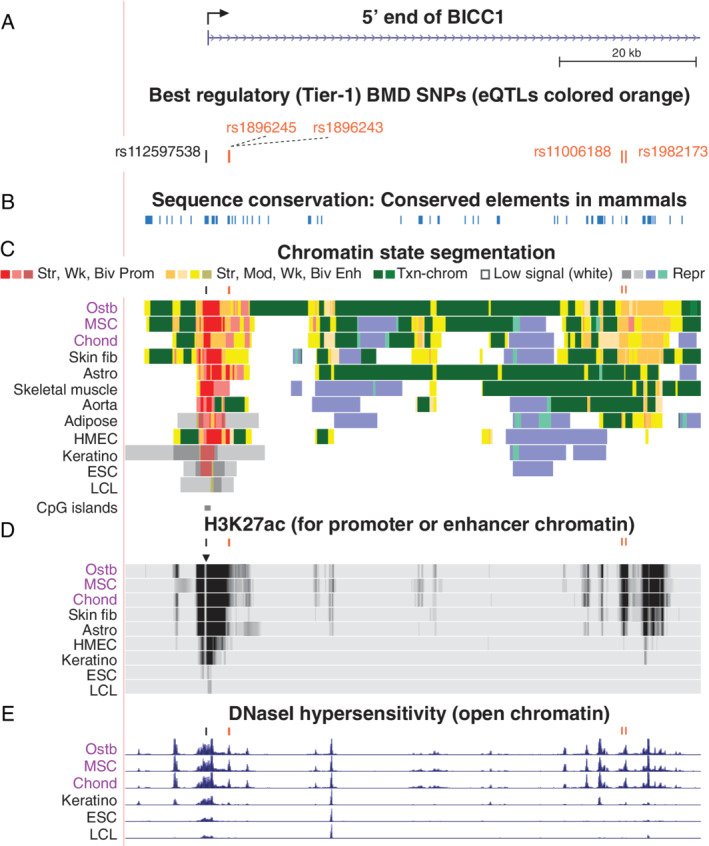
*BICC1*, which encodes an RNA‐binding protein involved in regulating Wnt signaling, has two clusters of Tier‐1 SNPs near its 5′ end. (*A*) An 84‐kb region around the *BICC1* TSS (chr10:60,260,940–60,345,012) with five Tier‐1 SNPs near the TSS (broken arrow) or downstream in intron 1. (*B*) DNA sequence conservation among placental mammals. (*C*) Roadmap‐derived chromatin state segmentation: strong (Str), moderate (Mod), weak (Wk), or bivalent (Biv; poised) promoter (Prom) or enhancer (Enh) chromatin; repressed (Repr) chromatin; or chromatin with the H3K36me3 mark of actively transcribed regions (Txn‐chrom); CpG islands, CpG‐rich regions. (*D*) H3K27ac enrichment profiles. (*E*) Profiles of open chromatin (DHS). Short bars above tracks in *C*, *D*, and *E* are positions of Tier‐1 SNPs. All tracks were visualized in the UCSC Genome Browser (hg19) and are aligned in this figure and Figs. 3, 4, 5, 6. Abbreviations: astro, astrocytes; chond, chondrocytes; DHS, deoxyribonuclease I hypersensitive site; fib, fibroblasts; HMEC, human mammary epithelial cells; ESC, embryonic stem cells; LCL, lymphoblastoid cell line; MSC, mesenchymal stem cells; NHEK, foreskin keratinocytes; Ostb, osteoblasts; SNP, single‐nucleotide polymorphism; TSS, transcription start site.

The five *BICC1* Tier‐1 SNPs were in two distinct clusters starting from 0.1 kilobase (kb) upstream of the *BICC1* transcription start site (TSS – 0.1 kb; rs112597538, rs1896245, and rs1896243) and within the central region of the 107‐kb intron 1 (rs11006188 and rs1982173; Fig. 2*A*). They were embedded in active promoter chromatin, mixed promoter/enhancer chromatin, or strong enhancer chromatin overlapping DHS in ostb, chond, and MSC (Fig. 2*C*–*E*), all of which display high expression of *BICC1* (Supplementary Fig. S3). rs112597538 overlapped a constitutive nucleosome‐depleted region seen as a ~0.17‐kb hole in the H3K27ac signal surrounding this SNP (triangle, Fig. 2*D*), which is indicative of strong nucleosome phasing due to TF binding. All five Tier‐1 SNPs are in moderate‐to‐high LD with each other (three are *r* ^2^ = 0.99 to 1; the other two are in perfect LD with each other and *r* ^2^ = 0.66 to the first three; Supplementary Fig. S7). rs112597538, rs1896245, and rs1982173 are located in sequences that are evolutionarily conserved among mammals (Fig. 2*B*), a trait enriched in noncoding transcription regulatory regions.

Four of the *BICC1* Tier‐1 SNPs were eQTLs for skeletal muscle and tibial artery (*p* = 1.5 × 10^−11^ to 7.7 × 10^−16^; no data were available for rs112597538 in the GTEx database^(^ ^23^ ^)^). The eQTLs that were in the proximal region of *BICC1* had opposite effect sizes from those in the downstream intron‐1 region for many tissues including skeletal muscle (Supplementary Table S7).^(^ ^23^ ^)^ Other Tier‐1 SNPs in *BICC1* might also influence expression in ostb given the large differences in enhancer chromatin profiles of ostb from those of skeletal muscle or aorta and the lack of available eQTL profiles for ostb. Importantly, the grouping of *BICC1* Tier‐1 SNPs according to the directionality of their eQTLs is consistent with their grouping by BMD‐increasing allele and with their locations in *BICC1* (Table 1; Fig. 2). Further support for the biological relevance of several or more of these Tier‐1 SNPs to osteoporosis is that all seven TFs or families of TFs are predicted to bind in an allele‐specific manner to the five *BICC1* Tier‐1 SNPs were related to the skeletal system (Supplementary Table S6).

### 3.3 *NPR3* has two intergenic BMD‐risk Tier‐1 SNPs upstream of a novel long intergenic noncoding RNA gene

**Fig 3:**
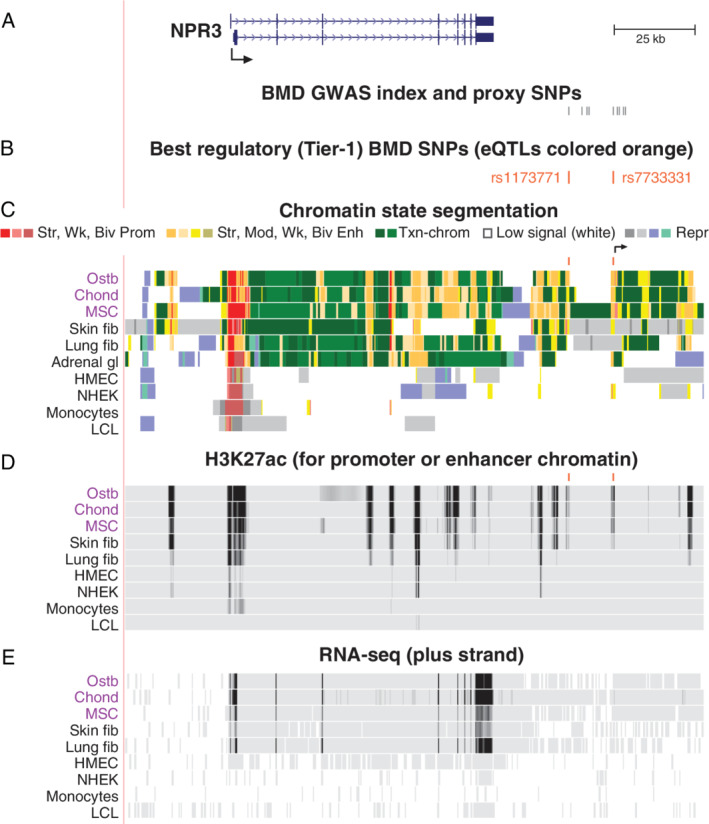
*NPR3*, which codes for a natriuretic peptide clearance receptor, is associated with two downstream Tier‐1 SNPs. (*A*) *NPR3* (chr5:32,678,200–32,856,800) and the BMD GWAS index and proxy SNPs (*r* ^2^ ≥ 0.8, EUR). (*B*) Tier‐1 SNPs. (*C*,*D*) Chromatin state segmentation and H3K27ac profiles as in Fig. 2. (*E*) Strand‐specific RNA‐seq (scale, 0–300). Broken arrow in *C*, ostb/chond/MSC‐specific TSS deduced from the CAGE signal for TSS; see Supplementary Fig. S8. Abbreviations: Adrenal gl, fetal adrenal gland (all other tissues are from adults); BMD, bone mineral density; chond, chondrocytes; GWAS, genome‐wide association study; MSC, mesenchymal stem cells; NHEK, skin keratinocytes; ostb, osteoblasts; SNP, single‐nucleotide polymorphism.

There were two Tier‐1 SNPs linked to the natriuretic peptide receptor 3 gene (*NPR3*; Table 1; Fig. 3*A* and *B*), which encodes a receptor that facilitates clearance of natriuretic peptides to regulate cell signaling.^(^ ^34^ ^)^ It is implicated in abnormal bone growth phenotypes in humans and mice^(^ ^29^ ^)^ consistent with its expression profile and enhancer chromatin profile (Fig. 3). Among various tissues, its highest expression is in aorta (Supplementary Fig. S4*B*). None of the nearby genes within its 1‐Mb neighborhood has a transcription profile like that of *NPR3* (Supplementary Table S7).

The *NPR3* Tier‐1 SNPs (rs1173771 and rs7733331) were downstream of the gene, in high LD (*r* ^2^ = 0.99), and 14 kb apart (Fig. 3*B*) and overlapped nucleosome‐depleted enhancer chromatin (Fig. 3*C* and *D*) in ostb, chond, and MSC (Supplementary Fig. S8). An ostb‐specific, MSC‐specific, and chond‐specific TSS for a gene encoding a novel long intergenic noncoding RNA (lincRNA) was found only 0.3 kb from rs7733331 by 5′ cap analysis gene expression (CAGE; Fig. 3*C*, arrow; Supplementary Fig. S8 *A*,*B*). The moderate‐to‐weak RNA‐seq signal from the plus‐strand for this unnamed lincRNA was not seen at low‐sensitivity settings (Fig. 3*E*), but was seen at higher sensitivity settings specifically in ostb, MSC, and especially chond (Supplementary Fig. S8 *C*). The *NPR3* SNPs were eQTLs for *NPR3* (*p* = 4.4 × 10^−41^ and 6.0 × 10^−39^) with positive effect sizes in tibial nerve. GTF2I is the TF predicted to bind with allele‐specificity to rs7733331, which is immediately upstream of the intergenic TSS (Table 1, Fig. 3*C*). This general TF binds specifically to initiator and E‐box DNA sequence elements in promoters^(^ ^31^ ^)^ and is implicated in osteoblastogenesis (Supplementary Table S6). Therefore, rs7733331, which overlaps this binding site, is an especially attractive candidate for a BMD‐regulatory SNP that might affect, in *cis*, enhancer‐associated *NPR3* transcription through a novel lincRNA gene.^(^ ^35^ ^)^

### 3.4 *LGR4* is associated with a BMD‐risk Tier‐1 SNP downstream of another protein‐coding gene

**Fig 4:**
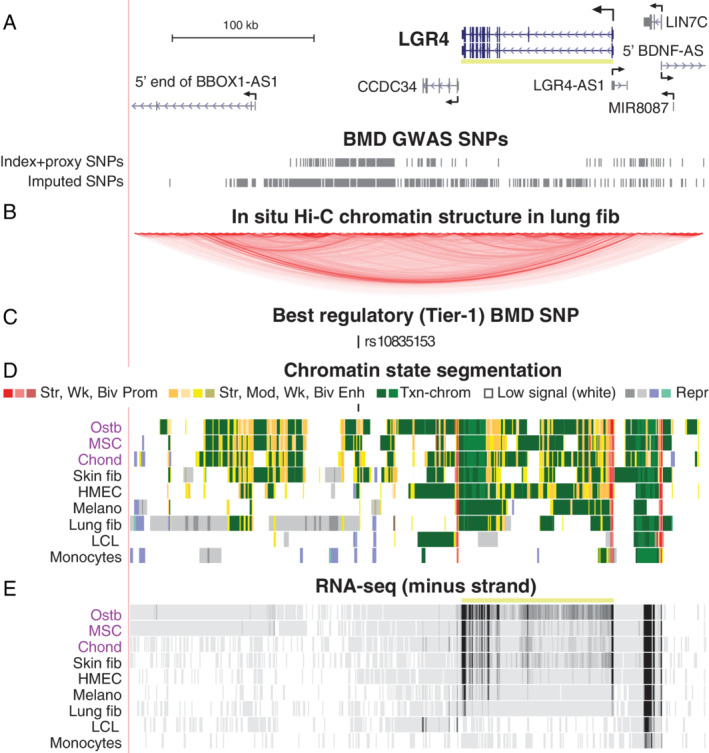
*LGR4*, which encodes a Wnt signaling receptor, has an intergenic Tier‐1 SNP far downstream of the gene. (*A*) *LGR4* and surrounding genes (chr11:27,154,000‐27,560,000) and the BMD GWAS index and proxy SNPs as well as imputed SNPs. (*B*) The Hi‐C track^(^ ^37^ ^)^ for chromatin folding for the fetal lung fib cell line IMR‐90; HMEC gave a similar profile (not shown). (*C*–*E*) As in Fig. 3 except that the minus‐strand signal is shown for RNA‐seq (scale, 0–100). Abbreviations: BMD, bone mineral density; fib, fibroblasts; GWAS, genome‐wide association study; HMEC, human mammary epithelial cells; Melano, foreskin melanocytes; SNP, single‐nucleotide polymorphism.

Leucine rich G protein‐coupled receptor 4 (*LGR4/GPR48;* Fig. 4*D*) was associated with one Tier‐1 SNP, rs10835153 (Table 1). This gene encodes a cell membrane receptor that can regulate Wnt signaling and is implicated in both embryonic bone development and postnatal bone remodeling.^(^ ^36^ ^)^ rs10835153 and most of the other BMD‐associated SNPs in this region were far downstream of *LGR4* and closer to the little‐studied *CCDC34* and *BBOX1*‐*AS1* genes than to the *LGR4* TSS (Fig. 4*C*), but neither of these genes is appreciably expressed in ostb, MSC, or chond (Supplementary Table S7). Chromatin interaction (Hi‐C) profiles^(^ ^37^ ^)^ for *LGR4*‐expressing lung fib and HMEC indicate that the *LGR4* promoter and the ostb enhancer chromatin overlapping *LGR4*'s intergenic Tier‐1 SNP, which are 180 kb apart, are in the same large chromatin loop (topologically associated domain, TAD) ending 2 to 13 kb upstream of the *LGR4* TSS (Fig. 4*B*). Ostb‐specific enhancer chromatin at the Tier‐1 SNP and ostb/MSC/chond/HMEC/fib enhancer chromatin further downstream of *LGR4* in this TAD are likely to upregulate the *LGR4* promoter because of the similar cell type‐specificity of their enhancer chromatin profiles and the *LGR4* transcription profile (Fig. 4*D*,*E*). The trait‐decreasing Alt allele of this Tier‐1 SNP is predicted to bind specifically to BPTF, the largest subunit of NURF, a nucleosome remodeling factor,^(^ ^31^ ^)^ which is associated with skeletal abnormalities (Supplementary Table S6).

### 3.5 *HMGA2* has three far‐upstream BMD‐risk Tier‐1 SNPs near a novel lincRNA gene

**Fig 5:**
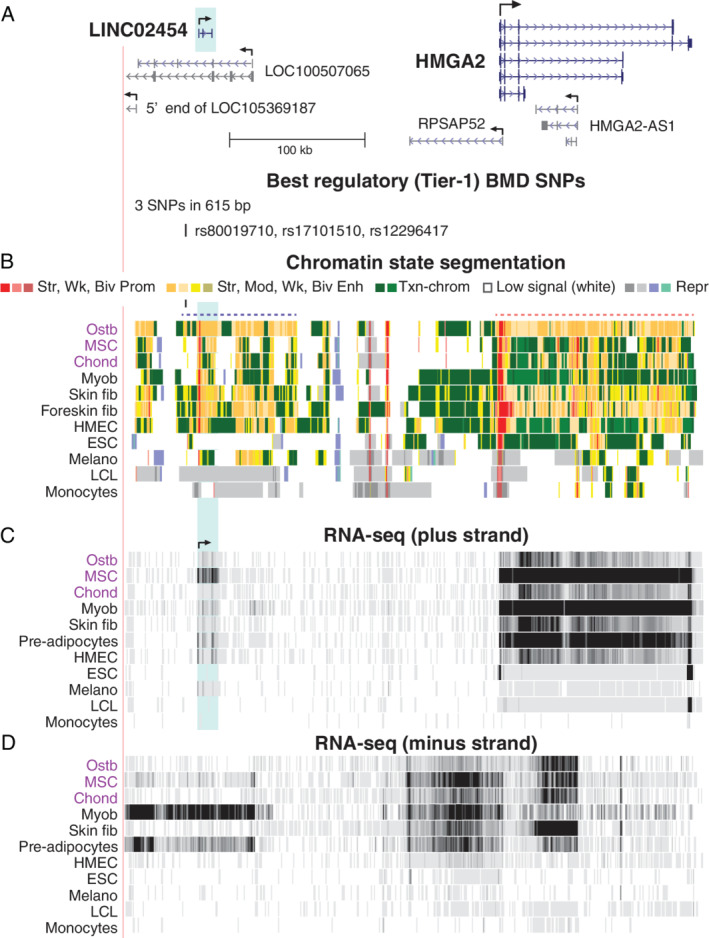
*HMGA2*, which encodes an enhanceosome protein, is associated with a cluster of far‐upstream intergenic Tier‐1 SNPs. (*A*) *HMGA2* and its upstream lincRNA genes and associated Tier‐1 SNPs are shown (chr12:65,942,326–66,365,457). (*B*–*D*) As in previous figures except that the RNA signal from both the plus and minus strands is shown (scales, 0–90 and 0–30, respectively). Dotted lines in *C* indicate super‐enhancers in ostb; blue highlighting, *LINC02454* region. Abbreviations: lincRNA, long intergenic noncoding RNA; Myob, myoblasts; SNP, single‐nucleotide polymorphism.

We identified *HMGA2*, which encodes high mobility group AT‐hook 2 protein and is part of multisubunit enhanceosome complexes, as a prime osteoporosis‐risk candidate. This was done by GO term enrichment for skeletal system genes rather than by preferential expression in ostb (Fig. 1). Despite *HMGA2*'s relationship to bone development^(^ ^38^, ^39^ ^)^ and its ostb‐associated super‐enhancer (a strong, unusually long enhancer) spanning the gene (Fig. 5*B*, dotted pink line), it is not preferentially expressed in ostb (Supplementary Table S5). However, even some BMD GWAS‐associated genes with negligible expression in the studied human ostb sample were linked to a limb phenotype in mouse models, possibly through effects on osteoclasts, MSC, chond, or on ostb or pre‐ostb in vivo but not in vitro (Supplementary Table S8). Similarly, although the steady‐state *HMGA2* RNA levels were low in the studied ostb, they were high in MSC, which can differentiate into ostb (Fig. 5*C*; Supplementary Table S5).

*HMGA2* was associated with three clustered intergenic Tier‐1 SNPs (rs80019710, rs17101510, and rs12296417; Table 1, Fig. 5*A*), 200 kb upstream of the *HMGA2* TSS in another super‐enhancer observed in ostb and skin fib (Fig. 5*B*, dotted purple line), both of which had low, but appreciable, levels of *HMGA2* RNA (Fig. 5*C*). Within this far upstream super‐enhancer are several lincRNA genes, one of which, *LINC02454*/*RP11‐221N13.3*, is 9 kb downstream of the Tier‐1 SNPs. Its TSS overlaps a constitutive binding site for the CTCF looping protein and is surrounded by ostb/myob/skin fib‐associated CTCF sites (Supplementary Fig. S9) that were not seen in cell cultures in which *HMGA2* was repressed. This suggests that the subregion containing the Tier‐1 SNP cluster is involved in expression‐related chromatin looping. Expression patterns of *LINC02454* and *RPSAP52* were correlated with those of their neighbor *HMGA2* (Fig. 5*C*,*D* and Supplementary Fig. S10). The noncoding RNA (ncRNA) gene *RPSAP52* can regulate *HMGA2* both posttranscriptionally by blocking *HMGA2*‐targeted miRNA activity and transcriptionally.^(^ ^40^ ^)^

The *HMGA2* far‐upstream Tier‐1 SNPs overlap predicted allele‐specific sites for TFs associated with bone or cartilage biology (Supplementary Table S6). Of special interest is HMGA1, a chromatin architectural protein similar to HMGA2, which is posttranscriptionally regulated by some of the same miRNAs that control *HMGA2* RNA^(^ ^41^ ^)^ and can upregulate Wnt signaling.^(^ ^42^ ^)^ Moreover, HMGA1 is critical for forming and maintaining bone and downregulates *miR‐196A‐2*, which targets *HMGA2* RNA in mouse embryonic fib.^(^ ^43^ ^)^

### 3.6 Slightly varying search parameters for BMD‐risk SNPs revealed likely osteoporosis‐risk regulatory SNPs for *DAAM2*

**Fig 6:**
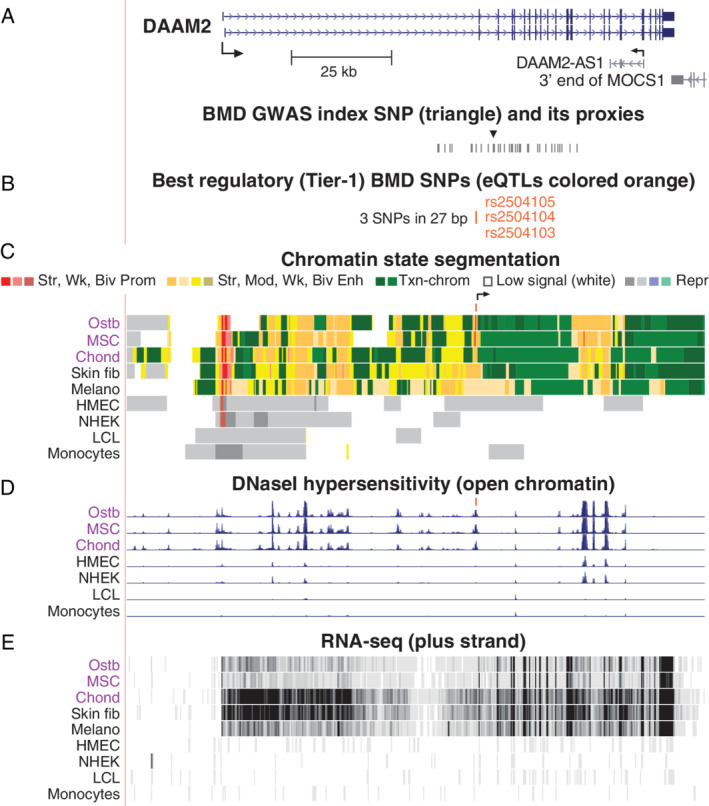
*DAAM2*, which codes for a Wnt signaling protein, has a cluster of three intragenic Tier‐1 SNPs. (*A*) *DAAM2* and its index and proxy SNPs from BMD GWAS (chr6:39,735,841–39,911,256). (*B*–*E*) As in previous figures except that the scale for RNA‐seq was 0–50. Abbreviations: BMD, bone mineral density; GWAS, genome‐wide association study; NHEK, skin keratinocytes; SNP, single‐nucleotide polymorphism.

By slightly relaxing one of our stringent requirements for defining Tier‐1 SNPs, we identified Disheveled associated activator of morphogenesis 2 (*DAAM2*) as a gene associated with highly credible candidates for BMD‐associated regulatory SNPs. In this modified protocol, we allowed BMD GWAS‐derived EnhPro SNPs to overlap a H3K27ac narrow peak in ostb and in no more than four (rather than the previous three) of the 12 other examined cell cultures from the Roadmap database. Three clustered SNPs (rs2504105, rs2504104, and rs2504103; Fig. 6 *A* and *B*) associated with *DAAM2* met this slightly modified requirement as well as the previous requirements for Tier‐1 SNPs (overlap of enhancer or promoter chromatin in ostb but no more than three of the other 12 cell types, overlap of a DHS peak in ostb, an allele‐specific TFBS prediction, and preferential ostb expression of its associated gene). *DAAM2* encodes a key regulator of the Wnt signaling pathway and is implicated in ostb mineralization as well as in bone resorption by murine osteoclasts,^(^ ^6^, ^44^ ^)^ consistent with its ratio of 21 for ostb/non‐ostb RNA levels (Supplementary Table S7).

We found that the eBMD index SNP rs2504101 highlighted in a previous report about *DAAM2* ^(^ ^6^ ^)^ (Fig. 6*A*, triangle) overlaps transcription‐type (H3K36me3‐enriched) chromatin in ostb, MSC, chond, and skin fib and does not overlap a DHS in any examined cell type. From 38 SNPs in high LD with this index SNP (*r* ^2^ > 0.8, EUR), we identified three Tier‐1 SNPs (rs2504105, rs2504104, and rs2504103; LD *r* ^2^ = 0.97; Supplementary Table S3) within a 27‐basepair (bp) subregion exhibiting enhancer/promoter chromatin preferentially in ostb, MSC, chond, and skin fib and overlapping an ostb DHS (Fig. 6*C*‐*E*). These intragenic SNPs were 0.4 kb upstream of a novel sense‐strand intronic TSS (CAGE signal; Fig. 6*C*, arrow) seen in the above cell types but not in cell cultures with repressed *DAAM2*. Moreover, they are eQTLs for tibial nerve (*p* = 8.4 × 10^−6^), a *DAAM2‐*expressing tissue (Supplementary Fig. S4 *D*). Therefore, one or more of the Tier‐1 SNPs in this cluster might upregulate *DAAM2* expression by increasing transcription of an intronic ncRNA at a cell type–specific enhancer. The much lower steady‐state levels of *DAAM2* RNA in ostb and MSC than in chond, despite their similar amounts of enhancer chromatin, could be due to posttranscriptional regulation.

Like rs11006188 in *BICC1*, the Ref allele of *DAAM2* Tier‐1 SNP rs2504105 is predicted to bind SMAD family TFs (Supplementary Table S6). In addition, the Alt allele of Tier‐1 SNP rs2504104 overlaps predicted binding sites for RUNX2, the master regulator of ostb lineage commitment,^(^ ^11^ ^)^ and CBFB, which can help stabilize Runx‐family proteins binding to DNA^(^ ^45^ ^)^ (Table 1). Last, the Alt allele of rs2504103 is predicted to bind nuclear factor I‐C (NFIC), which plays important roles in both tooth and bone development.^(^ ^46^ ^)^

### 3.7 Comparison of results from fine‐mapping and our detailed bioinformatic analysis

Using Bayesian fine‐mapping with the PAINTOR program,^(^ ^27^ ^)^ we examined ~2‐Mb regions centered on our Tier‐1 SNPs. With or without addition of epigenetic parameters (including Roadmap‐derived enhancer and promoter chromatin segmentation state), the fine‐mapping analysis found only two of our prioritized Tier‐1 SNPs, namely, *DAAM2*‐associated rs2504104 and rs2504104 with addition of epigenetic parameters and one *HMGA2*‐associated Tier‐1 SNP rs17101510; but, strangely, only with the baseline analysis (Supplementary Table S9). Only 32 of the SNPs were identified by this fine‐mapping approach as having a posterior probability ≥0.80 to be plausible causal, and none of these met our criteria for being an EnhPro SNP other than the above‐mentioned *DAAM2* and *HMGA2* Tier‐1 SNPs.

## 4 Discussion

We identified 14 high‐priority osteoporosis‐risk regulatory SNPs by using an unusual in‐depth bioinformatic approach relying on epigenomic and transcriptomic comparisons of ostb and heterologous cell cultures as well as prediction of allele‐specific binding of TFs that are important for bone biology (Fig. 1). These Tier‐1 SNPs, which had not been previously identified as candidates for causal regulatory SNPs for BMD, were associated with five skeletal system‐related genes. Three of these genes, *BICC1*, *LGR4*, and *DAAM2*, are involved in canonical Wnt signaling, a signaling pathway that plays critical roles in osteoblastogenesis and bone homeostasis.^(^ ^47^, ^48^, ^49^ ^)^ The other two genes are *NPR3*, and *HMGA2*, a mediator of natriuretic signaling and an essential component of enhanceosomes, respectively.^(^ ^29^, ^50^ ^)^ In comparison with our detailed bioinformatic prioritization of GWAS‐derived SNPs, fine‐mapping in the broad neighborhood of these genes was uninformative, as we found in a previous study.^(^ ^18^ ^)^ This may be attributable to fine‐mapping approaches giving insufficient weight to the input epigenetic data.

One of the most interesting genes for which we identified novel regulatory candidate SNPs is *BICC1*, which encodes a self‐polymerizing RNA‐binding protein that indirectly regulates Wnt signaling.^(^ ^33^ ^)^ This gene had two clusters of Tier‐1 SNPs near its 5′ end (Fig. 2). BICC1 is involved in osteoblastogenesis and polycystic kidney disease, in part, through its inhibition of posttranscriptional silencing of *PKD2* RNA by *miR‐17*.^(^ ^32^, ^51^, ^52^ ^)^ PKD2/PC2 and PKD1/PC1 form primary cilia that function as osteoporosis‐related mechanosensors in ostb (and probably osteocytes) and in epithelial kidney cells, and BICC1 has been detected in primary cilia in kidney cells.^(^ ^33^, ^53^, ^54^, ^55^ ^)^ Conditional inactivation of *Pkd2* in ostb/osteocytes of mice results in decreased levels of *Runx2* RNA and Wnt signaling along with the development of osteopenia.^(^ ^52^ ^)^ An important study by Mesner et al.^(^ ^32^ ^)^ implicating BICC1 in osteoporosis demonstrated that a heterozygous inactivating mutation in *Bicc1* in male mice led to low femoral BMD and that *Bicc1*‐deficent ostb had impaired differentiation in vitro which could be rescued by *Pkd2* overexpression.

*LGR4*, which had one associated Tier‐1 SNP (Fig. 4), encodes a bone‐related Wnt signaling receptor^(^ ^30^, ^56^ ^)^ and had been catalogued as one of many genes with an eBMD‐associated missense SNP (rs34804482) predicted to have a deleterious effect on protein structure.^(^ ^6^ ^)^ Moreover, Styrkarsdottir et al.^(^ ^57^ ^)^ found a rare nonsense mutation in the gene that is associated with low BMD, osteoporotic fractures in elderly individuals, electrolyte imbalance, and several types of cancer. In mice, homozygous deletion of *Lgr4* results in a low‐BMD phenotype, a strong delay in ostb differentiation and mineralization, and elevated numbers of osteoclasts.^(^ ^30^ ^)^ Osteoclasts and MSC precursors of ostb have also been implicated in the positive effects of LGR4 on bone formation and homeostasis from conditional mouse knockout models and in vitro studies.^(^ ^58^, ^59^ ^)^ However, the single Tier‐1 SNP that we found to be associated with *LGR4* was in enhancer chromatin in ostb but not in MSC. Epigenetic profiles of osteoclasts are not available but the finding of negligible levels of expression of *Lgr4* (and the mouse homologs of the other Tier‐1 associated genes) in cultured mouse osteoclasts and substantial levels in mouse ostb (Supplementary Fig. S6) suggests that the *LGR4* Tier‐1 SNP's regulatory role in osteoporosis risk is through ostb and possibly osteocytes.

Like *LGR4*, *DAAM2* encodes a regulator of canonical Wnt signaling implicated in the ostb and osteoclast lineages.^(^ ^6^, ^44^, ^60^ ^)^ Its 27‐bp cluster of three intragenic Tier‐1 SNPs, which are in perfect LD, may act through their overlapping enhancer/promoter chromatin in ostb, MSC, and chond and the novel intragenic TSS immediately downstream of these SNPs (Fig. 6*C*, arrow). Consistent with a role for *DAAM2* in inherited osteoporosis risk, an eBMD GWAS‐derived missense variant (rs201229313) in *DAAM2* that is probably deleterious had been identified previously by Morris et al.^(^ ^6^ ^)^ They also found that homozygous knockout of *Daam2* in mice decreases bone strength and increases cortical bone porosity. Moreover, inactivation of *DAAM2* in an ostb cell line using clustered regularly interspaced short palindromic repeats/CRISPR associated protein 9 impairs mineralization. We identified the *DAAM2* Tier‐1 SNPs by a very small change in one of the many requirements for defining Tier‐1 SNPs (Fig. 1) from less than four of the 12 non‐ostb–related cell cultures displaying the ostb‐associated H3K27ac peak overlapping the SNP to less than five of them. Therefore, by small variations of our protocol, future bioinformatic studies could uncover many more plausible BMD‐regulatory SNPs in addition to the 14 Tier‐1 SNPs determined in this study.

*NPR3* (*NPRC*) is involved not only in linear bone growth, bone turnover, and endochondral ossification,^(^ ^29^, ^61^, ^62^, ^63^ ^)^ but also in cardiovascular homeostasis, and renal cancer metastasis.^(^ ^29^, ^63^ ^)^ Its Tier‐1 SNPs (rs1173771 and rs7733331), which are in almost perfect LD, are upstream of another novel TSS observed preferentially in ostb and chond (Fig. 3*C* and Supplementary Fig. S8). *NPR3* encodes a transmembrane clearance receptor that opposes natriuretic peptide signaling through NPR1 and NPR2 receptors, which, unlike NPR3, contain guanylyl cyclase activity that is activated upon peptide binding. Enhancement of bone growth was found in patients with biallelic *NPR3* loss‐of‐function mutations or monoallelic gain‐of‐function mutations in *NPR2*.^(^ ^29^ ^)^ Osteocrin, a protein structurally resembling natriuretic peptides, binds specifically to NPR3 and is implicated in stimulating bone growth by limiting NPR3‐mediated clearance of natriuretic peptides.^(^ ^64^ ^)^ *NPR3* was associated with high bone density in a GWAS.^(^ ^65^ ^)^ *NPR3* is downregulated posttranscriptionally by microRNA 143 (*miR‐143*),^(^ ^66^ ^)^ whose host gene, *CARMN*, displays especially high expression in ostb.^(^ ^67^ ^)^ Both *NPR3*‐associated Tier‐1 SNPs have been previously identified as index SNPs in many blood pressure‐related GWAS, consistent with the role of *NPR3* also in the cardiovascular system.^(^ ^19^ ^)^ In addition, rs1173771 was associated with height in a GWAS.^(^ ^68^ ^)^

*HMGA2*, which has three clustered Tier‐1 SNPs far upstream of the gene (Fig. 5), was the only one of the five prioritized genes that was identified by its bone‐related GO association rather than by its ostb/non‐ostb expression ratio (Fig. 1). Some genes that impact osteoporosis risk through the ostb lineage may act at the pre‐ostb or osteocyte stages. Alternatively, some osteoporosis‐associated genes might be negatively associated with osteoblastogenesis. *HMGA2* may be an example of both phenomena. It encodes a chromosomal architectural and enhanceosomal protein implicated in negatively regulating the differentiation of bone marrow–derived MSC to ostb^(^ ^69^ ^)^ and is necessary for normal embryogenesis, including bone development.^(^ ^38^, ^39^ ^)^ It also is involved in carcinogenesis and metastasis,^(^ ^70^ ^)^ cell replication, and autophagy, which plays central roles in bone homeostasis through effects on ostb, osteocytes, and osteoclasts.^(^ ^71^ ^)^ *HMGA2* displays very low or negligible expression in normal postnatal tissues but is highly expressed in some mesoderm‐derived stem or progenitor cell types including MSC, myoblasts, and preadipocytes. It is much more weakly expressed in ostb, in accord with its negative effects on ostb formation from MSC.^(^ ^50^ ^)^ The need for tight control of *HMGA2* expression is evidenced by its unusually complex regulatory circuitry including the cancer‐associated *let‐7* miRNA and the *HMGA2*‐overlapping pseudogene *RPSAP52* and the anti‐sense gene *HMGA2‐AS1*.^(^ ^50^, ^72^ ^)^ Importantly, another ncRNA gene, *LINC02454*, which exhibits an *HMGA2‐*like expression pattern (Supplementary Fig. S10), is 9 kb downstream of the three Tier‐1 SNPs in an ostb‐associated super‐enhancer far from *HMGA2*. *LINC02454* was previously referenced only for its dysregulation in cancer cells.^(^ ^73^ ^)^ We propose that one or several of the *HMGA2*‐far upstream Tier‐1 SNPs help control expression of *LINC02454*, which, in turn, helps regulate transcription of *HMGA2* in MSC. The much lower steady‐state levels of *HMGA2* messenger RNA (mRNA) in ostb than in MSC despite the abundant enhancer chromatin within or upstream of the gene in ostb (Fig. 5) may be due to multiple miRNAs targeting *HMGA2*.^(^ ^72^ ^)^ Besides a role for *HMGA2* in negatively regulating MSC differentiation to ostb, it may play a protective role in the skeleton^(^ ^74^ ^)^ at later steps in differentiation of ostb to osteocytes and in osteocyte homeostasis by helping to induce autophagy.^(^ ^71^, ^75^, ^76^ ^)^

Like our five osteoporosis‐risk candidate genes, the TFs predicted to bind to the Tier‐1 SNP‐containing DNA sequences associated with these genes have special functions in the skeletal system (Supplementary Table S6). Further evidence implicating Tier‐1 SNPs as transcription‐regulatory variants comes from our finding that nine of the 14 SNPs for *BICC1*, *DAAM2*, or *NPR3* overlapped eQTLs for one or several tissues in which these genes are expressed. In the cases of *NPR3* and *DAAM2*, which had two to three Tier‐1 SNPs in high LD, only a single SNP at each of these loci may be the regulatory causal SNP. However, for *BICC1*, the dichotomy of the direction of effect sizes for tissue eQTLs, the LD structure for these SNPs, and the distribution of BMD‐increasing alleles for the two clusters of Tier‐1 SNPs (Supplementary Fig. S7) suggest that at least one Tier‐1 SNP in the TSS‐adjacent cluster and one in the 60‐kb downstream cluster are causal regulatory SNPs.

### 4.1 Limitations

A caveat in our identification of credible osteoporosis‐associated regulatory SNPs by epigenetic, transcription, and genetic analyses is that the application of one of our important criteria, the determination of allele‐specific TFBS, is difficult. Even when using a comprehensive database like TRANSFAC and manual curation, as we have done, such predictions have considerable numbers of false negatives and false positives. Our findings of the relevance to bone biology of the TFs corresponding to these TFBS predictions and the overlap of the TFBSs with enhancer or promoter chromatin, open chromatin, and narrow peaks of H3K27ac in ostb adds to the reliability of our TF binding predictions. However, TF binding can be influenced by the larger DNA sequence context, local and long‐range chromatin interactions, covalent modifications of TFs, TF protein–protein interactions, the cell type, and the physiological state of the cells. Specialized cell types like ostb usually have only very few genome‐wide studies of TF binding in vivo and lack publicly available eQTLs. Importantly, our detailed bioinformatics approach can empower in vivo testing for allele‐specific TF binding by providing credible predictions of allele‐specific TFBS for the careful experimental assays needed to detect the probably small effects of alternate alleles on expression of their associated genes.

Another limitation of the present study, which can be addressed in the future, is the lack of genome‐wide profiles for H3K4me1, H3K4me3, H3K27ac, open chromatin sites, and RNA‐seq for human osteoclasts and osteocytes. We did incorporate results from a published expression microarray study for mouse osteoclasts to show that none of the five prioritized genes obtained by using ostb‐based epigenomic and transcriptomic data were expressed in osteoclasts (Supplementary Figs S5 and S6). Therefore, future analyses of osteoclast data by our methods are not expected to affect our conclusions about ostb‐related Tier‐1 SNPs but rather, can add osteoclast‐related Tier‐1 SNPs.

## 5 Conclusion and Future Directions

Candidates for highly credible causal regulatory GWAS‐derived variants are more difficult to prioritize but are much more numerous than exonic variants affecting polypeptide structure. For our selection of 14 osteoporosis‐related regulatory SNPs associated with five genes from 38 BMD GWAS, we used stringent epigenomic and transcriptomic criteria. Our findings indicate the importance of not relying on computational methods like fine‐mapping without separately considering detailed bioinformatics data for epigenomics, transcriptomics, and TFBS prediction to evaluate the plausibility of candidate regulatory SNPs, as shown by our reevaluation of a previously highlighted BMD‐GWAS derived *DAAM2* SNP.^(^ ^6^ ^)^

The present study was intended to identify only a very small subset of particularly good ostb‐related candidates for transcription regulatory SNPs influencing the inherited risk to osteoporosis. The approach we took to prioritize osteoporosis‐associated genes and their Tier‐1 SNPs for future experimental studies can be extended for prioritization of additional candidates for regulatory BMD SNPs from the >3000 BMD GWAS index SNPs associated with >2000 genes. For example, preferential expression and epigenetic features in chond or bone marrow–derived MSC instead of ostb could be used. Additional epigenetic parameters, like cell type–specific regions of DNA hypomethylation or hypermethylation and higher‐order chromosome looping profiles, could be added as genomic profiles for bone‐relevant cells become available.

Elucidation of regulatory genetic variants strongly associated with disease susceptibility using epigenetic‐intensive strategies like ours can help find SNPs for subsequent experimental verification in cell culture and in animal models. Accurate and carefully designed testing of the biological effects of a GWAS‐derived candidate regulatory SNP is challenging. Differences in expression dependent on a GWAS SNP allele are expected to be small and may be highly dependent on the cell context and experimental methodology. Given the large amount of work to do these tests convincingly, it is essential to narrow the very large number of significant GWAS SNPs to the best candidates using extensive biologically based criteria as we have done in this bioinformatics study. Regulatory SNPs that are experimentally verified could be further tested for use as markers to identify at‐risk individuals for treatment or life‐style modifications. In addition, if the regulatory potential of novel ncRNA genes associated with candidate regulatory SNPs is confirmed, these genes can be studied further as to their suitability for development of pharmacological interventions.

## Conflict of Interest

All the authors state that they have no conflict of interest.

## Author Contributions

**Xiao Zhang:** Conceptualization; data curation; formal analysis; investigation; methodology; visualization; writing‐review & editing. **Hong Wen Deng:** Funding acquisition; project administration; resources; supervision; writing‐review & editing. **Hui Shen:** Funding acquisition; methodology; project administration; supervision; validation; writing‐review & editing. **Melanie Ehrlich:** Conceptualization; data curation; formal analysis; investigation; methodology; project administration; supervision; validation; visualization; writing‐original draft.

6

### Peer Review

The peer review history for this article is available at https://publons.com/publon/10.1002/jbm4.10481.

## Supporting information


**Appendix S1**: Supplementary Material.
